# Robot - assisted laparoscopic local recurrence resection after radical prostatectomy

**DOI:** 10.1590/S1677-5538.IBJU.2017.0503

**Published:** 2019

**Authors:** Fabio C. M. Torricelli, Paulo Afonso de Carvalho, Giuliano B. Guglielmetti, William C. Nahas, Rafael F. Coelho

**Affiliations:** 1 Serviço de Urologia, Hospital das Clínicas da Faculdade de Medicina da Universidade de São Paulo, São Paulo, SP, Brasil;; 2 Instituto do Cancer do Estado de Sao Paulo (ICESP), São Paulo, SP, Brasil;; 3 Hospital Israelita Albert Einstein, São Paulo, SP, Brasil

## Abstract

**Introduction and objective:**

Local prostate cancer recurrence is usually treated with salvage radiation (sRDT) with or without adjuvant therapy. However, surgical resection could be an option. We aim to present the surgical technique for robot - assisted laparoscopic resection prostate cancer local recurrence after radical prostatectomy (RP) and sRDT in 2 cases.

**Patients and method:**

First case depicts a 70 year - old man who underwent RP in 2001 and sRDT in 2004. Following adjuvant therapy, patient had biochemical recurrence. MRI showed a solid mass in the prostatic fossa close to vesicourethral anastomosis, measuring 2.1 cm and PET / CT revealed hyper caption significant uptake in the prostatic fossa. Second case is a 59 year - old man who underwent RP in 2010 and sRDT in 2011. Again, patient presented with biochemical recurrence. PET / CT showed hyper caption in the prostatic fossa. Biopsy conformed a prostate adenocarcinoma. Both patients underwent robot - assisted extended pelvic lymph nodes dissection and local recurrence resection. A standard 4 robotic arms port placement was utilized.

**Results:**

Both procedures were uneventfully performed in less than 3 hours and there were no complications. Pathological examination showed a prostate adenocarcinoma Gleason 7 and 8 in the first and second case, respectively; surgical margins and lymph nodes were negative. After 6 months of follow-up, continence was not affected and both patients presented with PSA < 0.15 ng / mL.

**Conclusion:**

Robot - assisted laparoscopic resection of prostate cancer local recurrence after RP and sRDT detected by PSMA PET / CT seems to be safe in experienced hands. It may postpone adjuvant therapy in selected cases.

## ARTICLE INFO

**Available at: http://www.intbrazjurol.com.br/video-section/20170503_Torricelli_et_al**

**Int Braz J Urol. 2019; 45 (video #3): 192-192**

